# Structural relationships among pair cohesion, tactical communication, and performance in doubles tennis: a structural equation modeling approach

**DOI:** 10.3389/fspor.2026.1920125

**Published:** 2026-10-26

**Authors:** Shiyuna Kazama, Hiroko Tanabe

**Affiliations:** Graduate School of Humanities and Human Sciences, Hokkaido University, Sapporo, Japan

**Keywords:** cognitive load, dyadic sport, group cohesion, perceptual congruence, structural equation modeling, tactical communication

## Abstract

**Introduction:**

Effective performance in doubles tennis requires partners to coordinate their actions within a shared competitive context. Although cohesion and communication are considered important for such coordination, less is known about whether within-dyad congruence in partners’ perceptions of cohesion is related to explicit tactical communication and performance. This study examined the relationships among within-dyad cohesion discrepancies, tactical communication, and performance in collegiate doubles tennis.

**Methods:**

Sixteen male collegiate tennis players formed 29 unique dyads. Pair cohesion was assessed using the Group Environment Questionnaire (GEQ), and within-dyad cohesion discrepancies were calculated as the absolute differences between partners’ ratings. Between-point verbal interactions were recorded and classified as tactical or non-tactical communication, and match performance was quantified using point, serve success, and return success rates. The relationships among cohesion discrepancies, tactical communication, and performance were examined using exploratory covariance-based structural equation modeling (CB-SEM), with PLS-SEM as a robustness check. Additional dependency-aware sensitivity analyses were conducted using match-level mixed-effects models and player-level multiple-membership models to account for dyads linked within matches and repeated player participation across dyads.

**Results:**

In the exploratory CB-SEM, greater within-dyad discrepancy in GI-S (Group Integration–Social) was associated with a lower tactical communication rate (*β* = −0.45, *p* = .047), while a higher tactical communication rate was associated with a lower point rate (*β* = −0.36, *p* = .012). However, the indirect effect of GI-S discrepancy on point rate through tactical communication was not statistically significant. PLS-SEM showed similar directions and magnitudes for these relationships, although the GI-S discrepancy–tactical communication path did not reach conventional statistical significance. In the dependency-aware analyses, the GI-S discrepancy–tactical communication association was not statistically significant in the match-level model and was not clearly supported in the player-level multiple-membership model. The tactical communication–point rate association remained negative in both analyses but was not clearly supported statistically.

**Discussion:**

The findings provide preliminary evidence that tactical communication in doubles tennis may be embedded within both relational and competitive contexts. The exploratory association between greater GI-S discrepancy and lower tactical communication raises the possibility that how similarly partners perceive their social integration may be relevant to explicit tactical coordination, although this association was not clearly supported after accounting for match- and player-level dependency. The consistently negative direction of the association between tactical communication and point rate further suggests that more frequent tactical communication does not necessarily reflect more effective coordination. Rather than supporting definitive relationships among cohesion, communication, and performance, these findings highlight the need to examine how relational perceptions, explicit tactical communication, and adaptation to unfolding competitive situations jointly shape dyadic coordination.

## 1 Introduction

Athletes must perform optimally under a wide range of volatile competitive conditions. Although performance enhancement efforts have traditionally emphasized physical and tactical preparation (1), accumulating evidence demonstrates that psychological factors play a critical role in shaping in-competition performance and match outcomes. Variables such as team cohesion, self-efficacy, and competitive anxiety consistently influence athletic performance across various sports contexts (2). Recent research has further linked motivation, emotion regulation, mental toughness (3), self-compassion (4), cognitive-load-induced mental fatigue (5), and sports anxiety (6) to performance-related outcomes. Similar associations have been documented in tennis, where psychological states modulate both physical performance and match success (7). Especially in interpersonal sporting environments, psychological dynamics emerge not only from individual traits but also from dyadic or group-level interactions. In team-based contexts, these interactions create emergent group-level characteristics that cannot be reduced solely to the sum of individual psychological or motor profiles (8). For instance, studies in women's basketball (9) and cheerleading (10) demonstrate that interpersonal impressions—such as perceived teammate effectiveness—significantly shape collective performance. Furthermore, positive interpersonal relationships among teammates foster a collective sense of efficacy, which ultimately enhances overall team functioning and performance outcomes (11). These findings suggest that interpersonal relationships may constitute a fundamental psychological resource underlying collective functioning in sport. Importantly, however, the functioning of such relationships may depend not only on how positively each athlete perceives the relationship, but also on the extent to which partners perceive that relationship similarly.

A central construct characterizing these collective dynamics is group cohesion, defined as a dynamic process reflecting the tendency for a group to stick together and remain united in the pursuit of its instrumental objectives and/or the satisfaction of member affective needs (12). Empirical studies using the Group Environment Questionnaire (GEQ) have demonstrated associations between cohesion and performance across various sports [e.g. (13, 14),]. Although the GEQ is completed by individual athletes, cohesion is conceptualized as a group-level construct, and multilevel examination of the GEQ has supported the relevance of both individual- and group-level components of cohesion (15). In a dyad, averaging the two partners’ scores can characterize their overall level of perceived cohesion; however, such averaging removes information about the extent to which their perceptions differ. Thus, the overall level of perceived cohesion and the degree of agreement between partners in their perceptions represent conceptually distinct aspects of a dyadic relationship. For example, two pairs may report the same mean level of cohesion while differing substantially in the extent to which the two partners agree in their perceptions. Such perceptual congruence may be particularly relevant in dyadic sports, in which two athletes must continuously coordinate with the same partner. Previous research on sporting dyads has shown that dissimilarity between partners in psychological characteristics can be associated with poorer relationship functioning (16), while research in team sport settings has similarly demonstrated the relevance of perceptual discrepancies between interacting members (17). These findings provide a conceptual basis for considering not only the level of perceived cohesion but also the degree of perceptual congruence between partners as a potentially meaningful relational characteristic. Accordingly, the present study explored within-dyad discrepancies in partners’ perceptions of cohesion as relational information that may not be captured by the pair-level mean alone.

Communication serves as an important mechanism through which these interpersonal relationships and collective dynamics are established, maintained, and operationalized. While athletes utilize both verbal and nonverbal channels, these forms of communication may serve partly distinct functions in interpersonal coordination. Nonverbal behaviors, such as interpersonal positioning and movement synchronization, can provide important information for interpersonal coordination and may themselves serve communicative functions (18). However, the meanings of nonverbal cues are context-dependent and require interpretation by the receiver, such that the specific tactical intentions underlying these behaviors may not always be unambiguously identifiable (19, 20). In contrast, verbal communication allows for rapid, explicit information exchange and mutual verification during intense competition (21). Through verbal exchanges, athletes can explicitly communicate and clarify tactical intentions and intended actions, thereby facilitating mutual understanding and the coordination of subsequent actions (22, 23). This function may be particularly important in dynamically changing sports contexts, where partners must make joint decisions and coordinate their subsequent actions within limited time. Thus, verbal communication between points provides an observable behavioral process through which doubles partners can explicitly coordinate their understanding and intended actions for subsequent play. However, despite its potential importance, it remains unclear how such explicit tactical coordination is related to the degree of congruence in partners’ perceptions of their dyadic relationship.

Despite the growing recognition of the psychological determinants of athletic performance, the precise behavioral pathways through which relational psychological characteristics are associated with objective performance via communication remain insufficiently specified. Crucially, conventional research focusing on team cohesion has predominantly relied on aggregated individual scores (e.g., calculating simple team averages) to examine its association with global performance outcomes. Such approaches fundamentally overlook the degree of alignment or discrepancy in cohesion perceptions within a pair, which represents relational information that cannot be captured by the pair-level mean alone. Consequently, the behavioral mechanisms through which perceptual discrepancies regarding cohesion are associated with explicit tactical communication within a dyad remain insufficiently examined. Beyond these conceptual gaps, these relationships remain insufficiently understood in dyadic sports such as doubles tennis, where performance emerges from tightly coupled interactions between two athletes. Moreover, relatively little is known about how cohesion perceptions and tactical communication are related to objective, rally-level performance outcomes rather than global match outcomes.

To address these empirical and methodological gaps, the present study aims to examine the relationships among perceptual congruence regarding cohesion, tactical communication, and real-time performance in doubles tennis. Specifically, we examine an exploratory structural model describing the relationships among within-dyad discrepancies in task and social cohesion, tactical communication, and rally-level performance outcomes. Previous research suggests that similarity or discrepancy between partners in psychological and interpersonal perceptions can be relevant to relationship functioning and coordination in sporting dyads and teams (16, 17). In addition, verbal communication enables athletes to explicitly share and clarify tactical intentions and intended actions, thereby facilitating interpersonal coordination during performance (22, 23). These findings provide a basis for expecting greater congruence in partners’ cohesion perceptions to be associated with more frequent tactical communication and, in turn, for tactical communication to be positively associated with performance. However, research in doubles tennis has shown that prior experience and shared expectations are associated with implicit coordination between partners (24). This suggests that effective coordination may not always depend on frequent explicit communication. Thus, the frequency of tactical communication should not necessarily be equated with the effectiveness of coordination. While acknowledging this alternative possibility, we retained and tested the following originally specified hypotheses:

H1: Pairs with smaller perceptual discrepancies (i.e., higher consensus) regarding perceived cohesion will exhibit a higher tactical communication rate.
H2: Tactical communication rate will be positively associated with objective, rally-level performance indices.
H3: Tactical communication will significantly mediate the relationship between within-dyad perceptual discrepancy in cohesion and rally-level performance.

## 2 Methods

### 2.1 Ethics statement

All participants provided written informed consent before participation. This study was approved by the institutional ethics committee of the Center for Experimental Research in Social Sciences, Hokkaido University (Approval No. R6-25) and adhered to the principles outlined in the Declaration of Helsinki.

### 2.2 Experimental protocols and measurements

#### 2.2.1 Participants

A total of 16 male collegiate tennis players participated in this study, forming 29 unique dyads. The participants were 20.7 ± 1.8 years old (mean ± SD), with 9.4 ± 2.9 years of tennis experience. Doubles-specific experience was not separately assessed. Some dyads had previously played doubles matches together; however, prior partner-specific playing and practice experience was not systematically recorded for all dyads. Because the study focused on dyadic interaction processes, participants were systematically recombined across sessions to create multiple unique pairings. Although the sample size was limited, strict inclusion criteria were applied to recruit male collegiate athletes competing at a comparable performance level, thereby reducing heterogeneity in technical skill and competitive experience, factors known to substantially influence communication patterns and performance outcomes in doubles tennis.

Some participants took part in multiple experimental sessions and were therefore included in multiple dyads. Across the 17 matches, 34 dyad observations were initially obtained; one was excluded because of an audio-recording failure, and four were excluded because the same partner combination had already appeared in an earlier session. Consequently, the final analytical sample comprised 29 unique dyads, with no repeated partner compositions. Detailed information on the participants’ tennis and doubles experience is presented in Table 1.

**Table 1:** Participant characteristics and competitive history.

Player ID	Age (years)	Tennis experience (years)	Doubles competitive history	Singles competitive history
P01	19	9	HS: Best 16 in Kyoto; College club: Quarterfinalist	HS: Champion in Kyoto public-school tournament; College club: Champion
P02	22	4	HS: None; College club: Second round	HS: None; College club: None
P03	23	9	HS: 3rd place in Asahikawa; College club: Quarterfinalist	HS: 1st place in Asahikawa; College club: Third round
P04	21	11	HS: Second round in All-Hokkaido; College club: Second round	HS: Second round in Sapporo Branch qualifiers; College club: None
P05	19	10	HS: Eliminated in district qualifiers; College club: Quarterfinalist	HS: Eliminated in district qualifiers; College club: Second round
P06	18	8	HS: Participated in prefectural competition; College club: Second round	HS: Prefectural qualifying finalist; College club: None
P07	18	9	HS: Best 8 in Hiroshima Prefecture Rookie Tournament; College club: Second round	HS: Best 32 in Hiroshima Prefecture; College club: None
P08	21	6	HS: Best 8 in Fukushima Prefecture; College club: First round	HS: Best 16 in Fukushima Prefecture; College club: None
P09	21	6	HS: Best 16 in city competition; College club: Second round	HS: Best 16 in city competition; College club: First round
P10	22	11	HS: Best 64 in Kanagawa Prefecture; College club: Champion	HS: Best 64 in Kanagawa Prefecture; College club: Third round
P11	21	12	HS: 1st place in Toyama Prefecture; College club: Runner-up	HS: 1st place in Toyama Prefecture; College club: 4th place
P12	24	17	HS: Best 8 in All-Japan competition; College club: Champion	HS: Best 32 in All-Japan competition; College club: Quarterfinalist
P13	21	9	HS: Best 16 in Shizuoka Prefectural Tournament; College club: Runner-up	HS: None; College club: None
P14	22	10	HS: None; College club: Second round	HS: None; College club: None
P15	21	11	HS: Best 32 in Mie Prefecture; College club: Quarterfinalist	HS: Best 64 in Mie Prefecture; College club: Quarterfinalist
P16	18	8	HS: 3rd place in Ishikawa Prefecture; College club: 3rd place	HS: 3rd place in Ishikawa Prefecture; College club: None

HS, high school. “College club” refers to intercollegiate competitions among university tennis clubs rather than varsity-level collegiate competitions. Tennis experience refers to overall tennis experience; doubles-specific experience was not separately assessed. Competitive history indicates the highest reported competitive achievement at the high-school and college-club levels.

Furthermore, the primary constructs of interest in the present study—pair cohesion, intra-pair cohesion discrepancy, communication content, and match outcome—were conceptualized and analyzed at the dyadic level, with each unique partner combination constituting the unit of analysis. However, because individual players participated in multiple dyads and opposing dyads were nested within matches, the dyadic observations could not be assumed to be fully independent. We therefore conducted additional analyses to account for these sources of dependency (see Section 2.4).

#### 2.2.2 Procedure

For each experimental session, participants were first assigned to a doubles pair and completed a warm-up. Immediately after the warm-up and just before the match, each participant completed the GEQ with reference to the partner with whom he was paired for that match. Each match was played as a single set using regular deuce scoring, with the first pair to reach six games declared the winner, regardless of the game difference. A total of 17 doubles matches were conducted.

Verbal interactions between partners were recorded throughout each match. In the present study, between-point communication was defined as verbal exchanges occurring after the completion of one point (i.e., after the rally had ended) and before the initiation of the subsequent point, typically within an interval of approximately 20 s. Conversations occurring during rallies were not included in the between-point communication analysis. Across the 17 matches, data were initially obtained from 34 dyad observations. One dyad was excluded because of an audio-recording failure, and four dyad observations were excluded because the same partner combination had already appeared in an earlier session. Consequently, 29 unique dyads were included in the final analyses.

#### 2.2.3 Data acquisition

Audio recordings were obtained using SYNCO G2A2 Pro lapel microphones with high noise-cancellation, maximum gain, and stereo settings at a sampling rate of 48 kHz. Video recordings were obtained from two fixed cameras positioned at opposite corners of the court to capture all points during each match.

Table 2 details the GEQ items. The GEQ is an 18-item self-report measure assessing cohesion in sport teams using a 9-point Likert scale. It consists of four subscales:

- ATG-S: Individual attractions to the group—social (5 items)
- GI-T: Group integration—task (5 items)
- ATG-T: Individual attractions to the group—task (4 items)
- GI-S: Group integration—social (4 items)

**Table 2:** Group environment questionnaire items adapted for the dyadic context.

Question No.	Question
1	自分は、このペアとの付き合いが楽しい。
	(I enjoy interacting with my partner.)
2	自分は、試合の時の自分のプレー時間に満足している。
	(I am satisfied with my playing time during matches.)
3	自分は、試合が終わってこのペアと会わなくなると寂しい。
	(I feel lonely when I do not see my partner after the match ends.)
4	自分は、このペアが持っている、試合に勝とうとする意欲に満足している。
	(I am satisfied with our pair's motivation to win matches.)
5	自分にとって、このペアは親しい友人である。
	(I consider my partner a close friend.)
6	自分は、このペアが自分のパフォーマンスを伸ばす機会を十分に与えてくれていると思う。
	(I believe that being part of this pair provides me with sufficient opportunities to improve my performance.)
7	自分は、他の人との付き合いより、このペアとの付き合いの方が楽しい。
	(I enjoy interacting with my partner more than interacting with other people.)
8	自分は、このペアのプレースタイルが気に入っている。
	(I like our pair's style of play.)
9	自分にとってこのペアでの活動は、自分（が所属している集団）の中で最も大切な関係の一つである。
	(For me, being part of this pair is one of the most important relationships within the groups to which I belong.)
10	このペアは、一致団結して目標を達成しようとしていると思う。
	(I think our pair is united in trying to achieve our goals.)
11	このペアは、自分と別々で出かけるよりも一緒に出掛けることを好むと思う。
	(I think my partner prefers going out together with me rather than separately.)
12	このペアは、試合で負けたり成績が思わしくないときは（たとえペア自身のせいでなくも）、責任を感じると思う。
	(I think my partner feels responsible when we lose a match or perform poorly, even when it is not their fault.)
13	このペアとは、ペア同士でよく飲み会などを企画する。
	(My partner and I often organize social gatherings together.)
14	このペアとは、目標が一致していると思う。
	(I think my partner and I share the same goals.)
15	このペアは、試合後も自分と一緒に過ごしたいと感じていると思う。
	(I think my partner wants to spend time with me after matches.)
16	このペアは、練習中にうまくできなかったら自分をサポートしてくれる。
	(My partner supports me when I do not perform well during practice.)
17	このペアとは、練習や試合以外でも仲が良い。
	(My partner and I have a good relationship outside of practice and matches.)
18	このペアは、試合や練習時にそれぞれの役割や責任などについて遠慮なく話し合う。
	(My partner and I openly discuss our respective roles and responsibilities during matches and practice.)

The Group Environment Questionnaire (GEQ) was adapted for the present dyadic context by replacing the term “team” with “pair.” The Japanese wording was reviewed and refined by two researchers to preserve the original intent and conceptual meaning of each item while ensuring natural and comprehensible expressions. Items that were reverse-worded in the original GEQ were reformulated as positively worded items. Table 2 presents the Japanese items used in the present study together with their English translations.

Following Carron's conceptualization (1985), the wording of the questionnaire was changed from “team” to “pairs,” which was refined by the two authors so that item meaning remained unchanged. The wording changes were limited to contextual adaptation for doubles tennis and did not alter the conceptual meaning of the original items. The internal consistency of each subscale of the GEQ was assessed using Cronbach's alpha coefficient. In this study, the alpha coefficients were.67 for ATG-T,.74 for ATG-S,.78 for GI-T, and.81 for GI-S. Although the internal consistency of the ATG-T subscale was slightly below the conventional benchmark of.70, this result is consistent with prior research using the GEQ. Previous studies have reported lower alpha coefficients for individual attractiveness subscales due to their limited number of items (12). Overall, these values were deemed acceptable for subsequent statistical analyses.

### 2.3 Data analysis

#### 2.3.1 Verbal communication coding and reliability

Verbal interactions occurring between points were manually transcribed by the first author. Each verbal exchange was then coded as either tactical or non-tactical communication. Tactical communication was defined as verbal exchanges concerning anticipated shots, player movements (self, partner, or opponents), court positioning, or strategic planning for the subsequent point. Representative examples of verbal exchanges coded as tactical communication are presented in Table 3 to illustrate the coding criteria.

**Table 3:** Representative examples of verbal exchanges coded as tactical communication.

Representative verbal exchanges	Tactical content
“Lob”, “high lob”, cross-court lob	shot
“Rally from the baseline and keep the net player out of the way”	shot
“Move the opponents around from the ne”，“Shake them up from the net”	motion
“Straight attack down the line”	shot
Move forward after serving; “play two-up”	motion
(Poaching volley) “Go for it”, “I’ll go”, “go when you can, poach when you can”	motion
“Stay” ，“Hold your position”，“Stay fixed”	motion
“Down-the-line coverage”, “down-the-line alert”, “forming a wall”	attention
“If the serve goes into the center, it becomes easier to poach”	motion
“They can rush the net”，"They can jam us at the net”	motion
“Make them hit ○○ instead” (force them to hit somewhere else)	shot

The table provides representative examples of verbal exchanges coded as tactical communication in the present study. The “Tactical content” column is provided to clarify the content represented by each example; these content descriptors were not analyzed as separate communication categories.

To assess the reliability of the tactical/non-tactical classification, a subset of the transcribed data was independently coded by a second trained rater. Inter-rater reliability was assessed using Cohen's kappa coefficient and indicated moderate agreement (*κ* = .60, 95% CI [.45,.74]), with an overall percent agreement of 92.7%. Coding discrepancies were resolved through discussion to reach a final consensus.

#### 2.3.2 Video data coding and performance metrics

Experimenters coded the outcome of each point, including the winning player and the shot type. Performance metrics were calculated as follows:

- Point rate: The proportion of points won
- Serve success rate: The proportion of successful serves during points where the pair was serving.
- Return success rate: The proportion of successful returns during points where the pair was receiving.

#### 2.3.3 Questionnaire data and cohesion metrics

Pair cohesion was assessed before each match using the four GEQ subscales (ATG-S, ATG-T, GI-S, GI-T). For each subscale, the mean of the two partners’ scores was first calculated to represent the overall level of perceived cohesion within the pair. These pair-level mean scores were examined in preliminary analyses but showed no significant associations with the communication or performance variables.

Given that averaging partners’ scores does not capture the extent to which the two partners differ in their perceptions of the same dyadic relationship, within-dyad perceptual discrepancy was subsequently examined as an exploratory relational metric. For each GEQ subscale, the discrepancy score was calculated as the absolute difference between the two partners’ subscale scores. The use of an absolute difference score provides a direction-independent index of the magnitude of discrepancy between two members of a dyad and has been used to operationalize dyadic discrepancy in previous research [e.g. (25),]. The discrepancy score was calculated as follows:

$$D_{i}=|S_{A,i}-S_{B,i}|,\;\;\;\;i\in \{\mathrm{ATG}-S,\;\mathrm{ATG}-T,\;\mathrm{GI}-S,\;\mathrm{GI}-T\}$$

where *D_i_* represents the within-dyad discrepancy for GEQ subscale *i*, and *S_A,i_* and *S_B,i_* represent players A and B's scores on that subscale, respectively. A value of zero indicated identical ratings between partners, whereas larger values indicated greater perceptual discrepancy. Thus, these scores were interpreted as indices of within-dyad discrepancy in perceived cohesion rather than as direct measures of dyadic cohesion itself.

Conversation-type rates were calculated at the dyad level for each match. For each conversation category, the number of points followed by a conversation classified into that category was divided by the total number of points played in that match and multiplied by 100. The conversation-type rate was calculated as follows:

$$R_{i}(\%)=\frac{N_{i}}{N_{total}}\times 100$$

where *R_i_* represents the rate of conversation type *i*, *N_i_* represents the number of points followed by conversation type *i*, *N_total_* represents the total number of points in the match, and *i* indicates either tactical or non-tactical communication.

The independent variables for preliminary analyses included conversation type (tactical and non-tactical) and match outcome. The dependent variables consisted of performance metrics (point rate, serve success rate, and return success rate) and within-dyad GEQ discrepancy scores. Missing data occurred in 19% of point rate values for tactical conversation and 4.8% for non-tactical conversation; no missing data were present for the GEQ discrepancy scores.

### 2.4 Statistical analysis

#### 2.4.1 Structural equation modeling (SEM)

SEM was employed to examine the hypothesized structural relationships linking within-dyad cohesion discrepancies, tactical communication, and performance outcomes. Given the relatively small sample size and the observational nature of the study, the SEM analysis was treated as exploratory. The hypothesized model, in which within-dyad cohesion discrepancies were related to performance outcomes through tactical communication, was tested using covariance-based structural equation modeling (CB-SEM). Analyses were performed in R using the *lavaan* package with maximum likelihood (ML) estimation. A nonparametric bootstrap procedure with 5,000 resamples was used to estimate standard errors and 95% confidence intervals (CIs), particularly for the indirect effect. Model fit was evaluated using the Comparative Fit Index (CFI), Root Mean Square Error of Approximation (RMSEA), and Standardized Root Mean Square Residual (SRMR), with conventional reference values of CFI >.95, RMSEA <.06, and SRMR <.08 used to aid interpretation of model fit. Given the limited sample size, model-fit indices and individual path estimates were interpreted cautiously rather than as definitive evidence of model adequacy.

#### 2.4.2 Robustness check by partial least squares SEM (PLS-SEM)

CB-SEM was retained as the primary structural modeling approach because the present study aimed to examine the hypothesized relationships among cohesion discrepancy, tactical communication, and performance within an integrated model. In addition, Partial Least Squares Structural Equation Modeling (PLS-SEM) was conducted as a robustness check to examine whether the direction and magnitude of the structural relationships observed in the CB-SEM were similar under an alternative estimation approach that is less reliant on large-sample assumptions. Consistent with the CB-SEM analysis, 5,000 bootstrap resamples were used to obtain estimates of uncertainty for the path coefficients and indirect effects. Consistent with the main analysis, 5,000 bootstrap resamples were applied to assess the significance of path coefficients and indirect effects. The PLS-SEM results were interpreted as a robustness check of the structural patterns observed in the exploratory CB-SEM rather than as an independent confirmatory test of the hypothesized model.

#### 2.4.3 Dependency-aware sensitivity analyses

Because individual players participated in multiple dyads and opposing dyads were linked within the same matches, the dyadic observations could not be assumed to be fully independent. Therefore, additional dependency-aware analyses were conducted to examine whether the two principal associations identified in the structural model—between GI-S discrepancy and tactical communication rate and between tactical communication rate and point rate—were retained after accounting for these sources of dependency. First, linear mixed-effects models were fitted with match as a random intercept to account for the clustering of opposing dyads within matches. The models were estimated in R using the *lme4* package with restricted maximum likelihood (REML) estimation. Separate models were estimated for GI-S discrepancy as a predictor of tactical communication rate and for tactical communication rate as a predictor of point rate. Second, because each dyad simultaneously comprised two players and individual players participated in multiple dyads, Bayesian multiple-membership models were fitted to account for repeated player participation. These models included the two players comprising each dyad as equal-weight multiple-membership random effects. Separate models were estimated for tactical communication rate as a function of GI-S discrepancy and for point rate as a function of tactical communication rate. The multiple-membership models were estimated in R using the brms package with four Markov chain Monte Carlo chains, 2,000 iterations per chain (1,000 warm-up iterations), and an adapt_delta value of.95. Model convergence was evaluated using R-hat values and the number of divergent transitions, and fixed-effect estimates were summarized using posterior means and 95% credible intervals. These dependency-aware analyses were treated as sensitivity analyses for evaluating the robustness of the principal associations identified in the exploratory structural model.

## 3 Results

### 3.1 Structural equation modeling (CB-SEM)

To examine the structural relationships among within-dyad cohesion discrepancies, tactical communication, and performance indices, an exploratory covariance-based structural equation modeling was estimated. The model yielded *χ*^2^(12) = 10.85, *p* = .542, CFI = 1.00, RMSEA = .000, SRMR = .112. Although the CFI and RMSEA indicated good fit according to conventional reference values, the SRMR exceeded the conventional cutoff criterion of.08, indicating that model fit was not consistently supported across indices. Accordingly, the model and its individual parameter estimates should be interpreted cautiously given the limited sample size. The estimated structural paths are presented in Figure 1.

**Figure 1:**
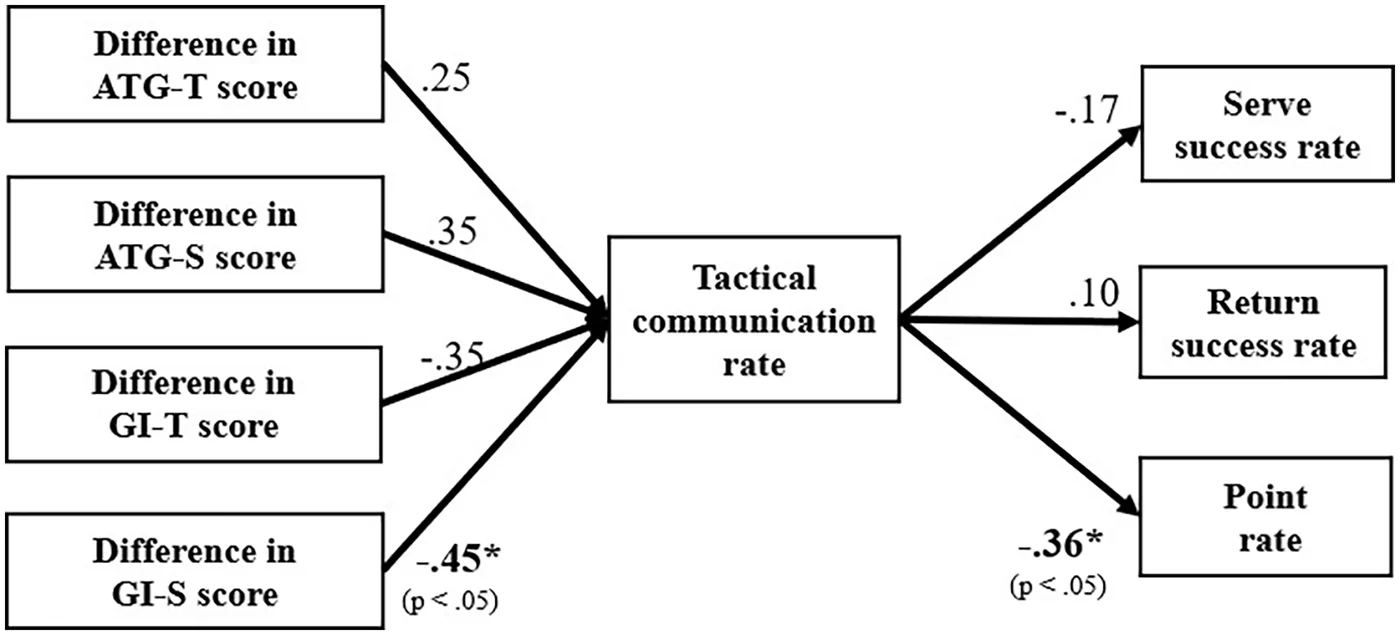
Exploratory CB-SEM of cohesion discrepancies, tactical communication, and performance. The four group environment questionnaire (GEQ) subscale discrepancy scores are shown on the left, tactical communication rate in the center, and performance indices (serve success rate, return success rate, and point rate) on the right. Values on the paths represent standardized path coefficients.

Regarding the structural paths, the within-dyad discrepancy in GI-S (Group Integration–Social) scores was negatively associated with tactical communication rate (*β* = −0.45, *p* = .047), whereas the path from discrepancies in ATG-T (Attraction to Group–Task; *β* = .25), ATG-S (Attraction to Group–Social; *β* = .35), and GI-T (Group Integration–Task; *β* = −.35) to tactical communication rate were not statistically significant. Regarding the paths from communication to performance, tactical communication rate was negatively associated with point rate (*β* = −0.36, *p* = .012), while its associations with serve success rate (*β* = −0.17) and return success rate (*β* = .10) were not statistically significant. The estimated indirect effect of GI-S discrepancy on point rate through tactical communication rate was *β* = .16. However, the 95% bootstrap confidence interval included zero (95% CI [−.015,.307]), indicating that the indirect effect was not statistically significant. Thus, although some individual paths reached statistical significance within the exploratory CB-SEM, the hypothesized indirect effect was not supported.

### 3.2 PLS-SEM analysis (robustness check)

The PLS-SEM results were largely consistent with the covariance-based SEM findings in terms of the direction and magnitude of the estimated paths. Specifically, the path from within-dyad discrepancy in GI-S scores to tactical communication rate showed a moderate negative association (*β* = −0.45, *p* = .052), although it did not reach conventional statistical significance. Tactical communication rate was negatively associated with point rate (*β* = −0.36, *p* = .010). The paths from tactical communication rate to serve success rate (*β* = −0.17) and return success rate (*β* = .10) remained not statistically significant.

Regarding the indirect effect, the estimated indirect effect of within-dyad GI-S discrepancy on point rate via tactical communication rate was *β* = 0.16. The 95% bootstrap confidence interval ranged from −0.005 to 0.345 and included zero, indicating that the indirect effect was not statistically significant. Overall, the PLS-SEM results showed a pattern similar to that observed in the exploratory CB-SEM in terms of the direction and magnitude of the estimated paths; however, the indirect effect was not supported in either analysis.

### 3.3 Dependency-aware sensitivity analyses

To account for the clustering of opposing dyads within matches, linear mixed-effects models were fitted with match as a random intercept. The association between GI-S discrepancy and tactical communication rate was not statistically significant (estimate = −0.0056, SE = 0.0046, 95% CI [−0.0151, 0.0039], *p* = .239). Tactical communication rate was negatively associated with point rate, although the association did not reach conventional statistical significance (estimate = −0.459, SE = 0.227, 95% CI [−0.927, 0.009], *p* = .054). In both models, the estimated match-level random-effect variance was zero, resulting in singular fits and indicating that no detectable between-match random-intercept variance was identified in these data.

Bayesian multiple-membership models were additionally fitted to account for the repeated participation of individual players across dyads. Both models showed satisfactory convergence, with no divergent transitions and maximum R-hat values of 1.009 or lower. The association between GI-S discrepancy and tactical communication rate was not clearly supported after accounting for repeated player participation (estimate = −0.0044, posterior SD = 0.0037, 95% credible interval [−0.0117, 0.0030]). Tactical communication rate showed a negative association with point rate, but its 95% credible interval included zero (estimate = −0.416, posterior SD = 0.256, 95% credible interval [−0.922, 0.094]). Thus, the statistically significant associations observed in the exploratory CB-SEM were not clearly supported after accounting for match- and player-level dependency, although the negative direction of the association between tactical communication rate and point rate was retained across the analyses.

## 4 Discussion

### 4.1 Overview of the main findings

Given the exploratory design and relatively limited sample size, the present findings should be interpreted cautiously as preliminary associations rather than evidence of definitive causal mechanisms. With respect to the proposed hypotheses, H1 received limited support: smaller within-dyad discrepancy in GI-S was associated with a higher tactical communication rate in the exploratory CB-SEM, whereas discrepancies in the other GEQ subscales were not significantly associated with tactical communication rate, and the GI-S association was not clearly supported in the dependency-aware analyses. H2 was not supported as hypothesized: tactical communication rate was negatively, rather than positively, associated with point rate in the exploratory CB-SEM, while its associations with serve success rate and return success rate were not statistically significant. Although the negative direction of the association with point rate was retained in the dependency-aware analyses, it was not clearly supported statistically. H3 was not supported because the indirect effect of GI-S discrepancy on point rate through tactical communication was not statistically significant.

The PLS-SEM yielded similar directions and magnitudes for these structural paths, although the path from GI-S discrepancy to tactical communication did not reach conventional statistical significance. Importantly, the additional dependency-aware sensitivity analyses provided more limited support for these associations. In the match-level mixed-effects analyses, the association between GI-S discrepancy and tactical communication was not statistically significant, and the negative association between tactical communication and point rate did not reach conventional statistical significance. Similarly, the player-level multiple-membership analyses did not provide clear evidence for either association, although the negative direction of the association between tactical communication and point rate was retained.

Overall, these findings suggest that the relationships among cohesion perceptions, tactical communication, and performance may be partly embedded within the broader competitive context in which doubles interactions occur. The attenuation of the associations after accounting for match- and player-level dependency does not, by itself, establish that the patterns observed in the exploratory SEM were irrelevant; rather, it suggests that they may partly reflect match-level conditions and characteristics of the individual players composing each dyad. In actual doubles competition, communication and performance emerge within such interdependent contexts rather than in isolation. Accordingly, the contrast between the exploratory structural models and the dependency-aware analyses may help distinguish associations observed within the naturally embedded competitive environment from those that remain after accounting for match- and player-level dependency. However, the present analyses were not designed to identify which specific match- or player-level factors accounted for the attenuation of these associations, and the findings should therefore be regarded as hypothesis-generating.

### 4.2 Cohesion congruence and tactical communication

The exploratory CB-SEM identified a negative association between within-dyad discrepancy in GI-S (Group Integration–Social) and tactical communication rate, whereas no statistically significant associations were observed for discrepancies in the other GEQ subscales. This association was not statistically significant in the match-level mixed-effects analysis, although no detectable between-match random-intercept variance was identified. The association was also not clearly supported in the multiple-membership analysis accounting for repeated player participation across dyads. Thus, the GI-S association should be interpreted as an exploratory pattern rather than a robust relationship independent of match- and player-level dependency. In addition, no substantial difference was observed in total GEQ scores between winning and losing pairs (winning pairs: 130 ± 15.6; losing pairs: 130 ± 16.7). Taken together, the present findings provide limited evidence for a direct relationship between cohesion and communication or performance, while leaving open the possibility that specific aspects of partners’ cohesion perceptions may be relevant within the broader competitive context.

Nevertheless, it is noteworthy that GI-S was the only GEQ subscale for which a statistically significant path to tactical communication emerged in the exploratory CB-SEM. GI-S reflects members’ perceptions of the social integration of the group, whereas the tactical communication examined in the present study concerned task-oriented content, including anticipated shots, movement, and strategic planning. The emergence of a social rather than task-related cohesion dimension is therefore of potential theoretical interest. One possible interpretation is that explicit tactical exchanges may be shaped not only by task-related perceptions but also by how similarly partners perceive the social relationship within the dyad. Previous research in sporting dyads has shown that dissimilarity between partners in psychological characteristics can be associated with poorer relationship functioning (16), while research in team sport settings has similarly demonstrated the relevance of discrepancies in interpersonal perceptions (17). From this perspective, the GI-S pattern observed in the exploratory SEM raises the possibility that the social-relational context of a dyad may be relevant to how partners engage in explicit tactical communication. However, because this association was not consistently supported after accounting for the dependency structure of the data, this interpretation remains tentative and hypothesis-generating.

The distinction between the overall level of perceived cohesion and within-dyad discrepancy may nevertheless provide a useful perspective for future research. A pair-level mean represents the overall level of cohesion perceived by the two partners, whereas a discrepancy score captures the extent to which their perceptions of the same dyadic relationship differ. Thus, two dyads can have similar mean levels of cohesion while differing substantially in the degree of perceptual congruence between partners. The absence of a clear difference in total GEQ scores between winning and losing pairs therefore does not necessarily imply that partners’ relational perceptions are irrelevant to dyadic interaction; rather, aggregate cohesion levels and within-dyad perceptual discrepancies represent distinct aspects of the dyadic relationship. At the same time, the present findings do not establish that perceptual congruence is more important than the overall level of cohesion. Given the exploratory nature of the discrepancy analysis and the limited sample size, future studies with larger samples should retain both partners’ original ratings and examine their joint effects using dyadic analytical approaches that can distinguish the effects of perceptual congruence from the underlying levels of each partner's cohesion perceptions.

### 4.3 Tactical communication and performance

Across the exploratory structural models, higher tactical communication rates were associated with lower point rates. Although this association did not reach conventional statistical significance in the match-level dependency-aware analysis and was not clearly supported in the player-level multiple-membership analysis, the direction of the association remained negative across the analytical approaches. Thus, the present findings do not establish that greater tactical communication leads to poorer performance, but they suggest that tactical communication and performance may be negatively related within the heterogeneous and interdependent contexts of actual doubles competition. Previous research has emphasized that effective team communication cannot be characterized solely by the amount of information exchanged, but also depends on how communication functions within ongoing coordination processes (23, 26). Accordingly, the present pattern suggests that a higher frequency of explicit tactical exchanges should not necessarily be interpreted as an indicator of more effective coordination.

One possible explanation for the observed negative pattern concerns the cognitive demands associated with explicit tactical information processing immediately before play. Doubles tennis requires players to rapidly select and execute actions while responding to continuously changing information. Previous research has shown that complex executive demands and concurrent cognitive and physical tasks can increase cognitive load and interfere with performance (27, 28). In the present study, tactical communication included specific discussions concerning anticipated shots, player movements, and strategic planning immediately before the subsequent point. It is therefore possible that, under some competitive conditions, extensive tactical information exchange immediately before play increases the cognitive demands placed on players and interferes with rapid or relatively automatic action execution. A related possibility is that explicitly discussing an anticipated tactical scenario may facilitate preparation for a particular course of action, while making rapid adjustment more demanding when the actual rally unfolds differently from what was anticipated. Basic research on task switching has consistently demonstrated performance costs when individuals must shift from a previously prepared or activated task set to another, reflecting both reconfiguration demands and interference from the preceding task set (29, 30). Thus, when the unfolding rally deviates from the tactical scenario discussed between points, players may need to update or switch from the prepared action plan to an alternative response, potentially delaying adaptive action. However, cognitive load was not directly measured in the present study, and this explanation should therefore be regarded as one possible mechanism rather than a demonstrated account of the observed association.

An alternative, and equally plausible, interpretation is that increased tactical communication reflects a response to difficulties already occurring during the match. Pairs experiencing unsuccessful points or tactical problems may engage in more explicit discussion to adjust positioning, shot selection, or strategic plans. From this perspective, poorer performance may elicit tactical communication rather than tactical communication producing poorer performance. This possibility is particularly relevant because communication and performance in actual doubles matches are dynamically embedded within the unfolding competitive context, including the actions of the opposing pair and the evolving state of the match. The present observational design cannot distinguish these temporal or causal directions. Future studies using point-by-point sequential or time-series analyses could examine whether tactical communication predicts subsequent performance, whether performance difficulties increase subsequent communication, or whether these processes operate bidirectionally.

Tactical communication rate was not significantly associated with serve success rate or return success rate, whereas the negative association was observed specifically for point rate in the exploratory structural models. One possible explanation is that serving and returning represent more constrained components of play, whereas the subsequent rally requires continuous adaptation to the actions and positioning of both partners and opponents. Tactical information exchanged between points may therefore be more closely related to performance during the broader, dynamically evolving point than to the success of an individual serve or return. It is also possible that some well-coordinated dyads require less explicit verbal exchange because aspects of their coordination have become more implicit or anticipatory. From this perspective, a lower frequency of tactical communication would not necessarily indicate poorer coordination. These possibilities remain speculative, however, and future studies combining verbal communication with nonverbal coordination and point-by-point performance dynamics are needed to determine how explicit communication functions within doubles competition.

### 4.4 Theoretical and practical implications

The present findings provide preliminary support for viewing tactical communication in doubles tennis as a process embedded within both relational and competitive contexts. Although the association between within-dyad GI-S discrepancy and tactical communication was identified only in the exploratory CB-SEM and was not clearly supported by the dependency-aware analyses, the emergence of a social rather than task-related cohesion dimension is theoretically noteworthy. This pattern raises the possibility that explicit tactical coordination may be related not only to task-related factors but also to how similarly partners perceive their social integration within the dyad. More broadly, tactical communication in dyadic sports may therefore be usefully conceptualized not solely as the transfer of tactical information, but as a coordination process that occurs within an interpersonal relationship and an unfolding competitive context. Rather than indicating that perceptual congruence is inherently more important than the overall level of cohesion, the present findings suggest that the relational context in which tactical communication occurs may warrant consideration alongside the content and frequency of communication.

From a practical perspective, these findings suggest the potential value of distinguishing and jointly training several components of dyadic coordination: partners’ relational perceptions, the content and timing of explicit tactical communication, and flexible adaptation to the unfolding competitive situation. Tactical communication may facilitate coordination by allowing partners to explicitly share intended actions and strategies, but extensive or highly specific pre-point planning may also impose cognitive demands when the subsequent play deviates from the anticipated scenario and requires rapid updating of the prepared action plan. Accordingly, training may benefit not simply from encouraging more or less communication, but from helping pairs develop when and how to communicate tactical information efficiently, while also maintaining the capacity to flexibly adapt when anticipated and actual situations diverge. Although these practical implications remain speculative and require experimental verification, such an approach may provide a useful framework for considering how relational, communicative, and adaptive processes jointly contribute to coordination in doubles tennis.

### 4.5 Limitations and future directions

Several limitations should be acknowledged. First, because the present study employed an observational, cross-sectional design, causal relationships cannot be directly established. In particular, the observed association between tactical communication and performance does not establish whether tactical communication influences subsequent performance or whether poorer performance prompts greater tactical communication between partners. Second, the participants consisted exclusively of male collegiate tennis players, which may limit the generalizability of the findings to other populations, skill levels, or sports contexts. Third, the relatively small sample size (*N* = 29 dyads) limited statistical power and the stability of model estimates, particularly for detecting indirect effects. In addition, the dyadic observations were not fully independent because individual players participated in multiple dyads and opposing dyads were linked within matches. Although dependency-aware sensitivity analyses were conducted to account for these structures, the limited sample size constrained the complexity of the models that could be estimated and the precision with which player- and match-level effects could be distinguished. Accordingly, the structural relationships identified in the exploratory SEM require replication in larger samples that permit more comprehensive multilevel or dyadic modeling. Fourth, the present study focused solely on verbal communication and did not consider nonverbal forms of interpersonal coordination, such as positioning, movement synchronization, gestures, or other behavioral cues.

Fifth, prior partner-specific playing and practice experience was not systematically assessed. Although some dyads were known to have previously played doubles matches together, information regarding the presence, frequency, and duration of prior experience with the specific partner was not available for all dyads. Such prior familiarity may have influenced partners’ perceptions of cohesion and their communication patterns. Future studies should systematically assess and account for partner-specific playing and practice experience when examining interpersonal processes in doubles tennis. Sixth, although the GEQ items were adapted to the dyadic context by replacing “team” with “pair” and the Japanese wording was reviewed and refined to preserve the conceptual meaning of the original items, the psychometric validity of this dyadic adaptation was not independently established in the present sample. Accordingly, findings based on the adapted GEQ should be interpreted cautiously, and future research should further examine its validity for assessing cohesion perceptions in sporting dyads. In addition, within-dyad discrepancies in perceived cohesion were operationalized using absolute difference scores between partners’ GEQ ratings. Although this approach provides a straightforward index of the magnitude of perceptual incongruence, difference scores collapse the two partners’ original ratings into a single value and therefore do not distinguish between different combinations of cohesion levels that yield the same degree of discrepancy. More generally, an association involving a difference score does not necessarily demonstrate that discrepancy itself accounts for the outcome, because the association may instead reflect the underlying level of one or both component variables (31). Accordingly, the present findings do not establish that perceptual discrepancy itself, rather than the underlying cohesion levels of the two partners, accounts for the observed associations, and the discrepancy-related findings should therefore be regarded as exploratory. Future studies with larger samples should retain both partners’ original ratings and examine their joint effects using approaches such as polynomial regression and dyadic response surface analysis, which enable the effects of perceptual congruence and the underlying levels of both partners’ perceptions to be examined simultaneously (32).

Future research should also incorporate point-by-point time-series or sequential approaches to examine how communication and performance dynamically influence one another across successive points. In particular, linking the tactical content discussed between points to the events occurring during the subsequent point would make it possible to examine whether performance depends on the correspondence between anticipated and actual play, and how players adapt when the unfolding situation deviates from the previously discussed tactical plan. Future studies should further integrate verbal communication with nonverbal forms of interpersonal coordination, such as positioning, movement synchronization, and gestures, to provide a more comprehensive account of how doubles partners coordinate their actions during competition.

## Data Availability

The raw data supporting the conclusions of this article will be made available by the authors, without undue reservation.

## References

[1] Baker J Horton S Robertson-Wilson J Wall M. Nurturing sport expertise: factors influencing the development of elite athlete. J Sports Sci Med. (2003) 2(1):1–9. 24616603 PMC3937568

[2] Lochbaum M Stoner E Hefner T Cooper S Lane AM Terry PC. Sport psychology and performance meta-analyses: a systematic review of the literature. PLoS ONE. (2022) 17(2):e0263408. 10.1371/journal.pone.0263408 35171944 PMC8849618

[3] Rossi C Roklicer R Tubic T Bianco A Gentile A Manojlovic M. The role of psychological factors in judo: a systematic review. Int J Environ Res Public Health. (2022) 19(4):2093. 10.3390/ijerph19042093 35206281 PMC8871700

[4] Doorley JD Kashdan TB Weppner CH Glass CR. The effects of self-compassion on daily emotion regulation and performance rebound among college athletes: comparisons with confidence, grit, and hope. Psychol Sport Exerc. (2022) 58:102081. 10.1016/j.psychsport.2021.102081

[5] Sun H Soh KG Roslan S Wazir MRWN Soh KL. Does mental fatigue affect skilled performance in athletes? A systematic review. PLoS One. (2021) 16(10):e0258307. 10.1371/journal.pone.0258307 34648555 PMC8516214

[6] Bukhari FK Fahd S Tahira R Yaseen M. Impact of sports anxiety on sports performance of players. Pak J Humanit Soc Sci. (2021) 9(3):581–6. 10.52131/pjhss.2021.0903.0163

[7] Song Y Lin H Wang B Yang M. Factors and measures affecting players’ psychology in tennis. JSSHL. (2024) 7(3):106–11. 10.53469/jsshl.2024.07(03).14

[8] Forsyth DR. Group Dynamics (7th ed.). Boston, MA: Cengage Learning (2019).

[9] Brynzak S Putrov S Olena O Ruslan M Kostenko M Prima A. Consideration of psychological compatibility of female athletes in maintaining psychological climate of women’s basketball teams. J Phys Educ Sport. (2021) 21(1):343–51. 10.7752/jpes.2021.01032

[10] Habeeb CM Stephen SA Eklund RC. Team efficacy profiles: congruence predicts objective performance of athlete pairs. J Sport Exerc Psychol. (2024) 46(1):22–33. 10.1123/jsep.2023-0044 38167218

[11] Jowett S Shanmugam V Caccoulis S. Collective efficacy as a mediator of the association between interpersonal relationships and athlete satisfaction in team sports. Int J Sport Exerc Psychol. (2012) 10(1):66–78. 10.1080/1612197X.2012.645127

[12] Carron AV Widmeyer WN Brawley LR. The development of an instrument to assess cohesion in sport teams: the group environment questionnaire. J Sport Psychol. (1985) 7(3):244–66. 10.1123/jsp.7.3.244

[13] Choudhary PK Dubey S Sahu DKK. Comparative evaluation of group cohesion among team sports. J Sports Sci Nutr. (2022) 3(2):194–8. 10.33545/27077012.2022.v3.i2c.122

[14] Koushik KVS Sanjay P Senthuran S Jadhav A Kolli ET. The role of sports psychology in enhancing team cohesion and performance. TPM. (2025) 32(S2):2236–42.

[15] Whitton SM Fletcher RB. The group environment questionnaire: a multilevel confirmatory factor analysis. Small Gr Res. (2014) 45(1):68–88. 10.1177/1046496413511121

[16] Jackson B Harwood CG Grove JR. On the same page in sporting dyads: does dissimilarity on 2× 2 achievement goal constructs impair relationship functioning? J Sport Exerc Psychol. (2010) 32(6):805–27. 10.1123/jsep.32.6.805 21282839

[17] Light Shields DL Gardner DE Light Bredemeier BJ Bostro A. The relationship between leadership behaviors and group cohesion in team sports. J Psychol. (1997) 131(2):196–210. 10.1080/00223989709601964

[18] Vesper C Sevdalis V. Informing, coordinating, and performing: a perspective on functions of sensorimotor communication. Front Hum Neurosci. (2020) 14:168. 10.3389/fnhum.2020.00168 32528263 PMC7264104

[19] Hall JA Horgan TG Murphy NA. Nonverbal communication. Annu Rev Psychol. (2019) 70:271–94. 10.1146/annurev-psych-010418-103145 30256720

[20] Furley P. The nature and culture of nonverbal behavior in sports: theory, methodology, and a review of the literature. Int Rev Sport Exerc Psychol. (2023) 16(1):448–73. 10.1080/1750984X.2021.1894594

[21] Uchil A. Exploring the multifaceted nature of verbal communication. In: Sahay M Amin P, editors. A Textbook of Communication, Media and Society. New Delhi: Wisdom Press (2022). p. 16–25.

[22] Lausic D Tennebaum G Eccles D Jeong A Johnson T. Intrateam communication and performance in doubles tennis. Res Q Exerc Sport. (2009) 80(2):281–90. 10.1080/02701367.2009.10599563 19650394

[23] LeCouteur A Feo R. Real-time communication during play: analysis of team-mates’ talk and interaction. Psychol Sport Exerc. (2011) 12(2):124–34. 10.1016/j.psychsport.2010.07.003

[24] Blickensderfer EL Reynolds R Salas E Cannon-Bowers JA. Shared expectations and implicit coordination in tennis doubles teams. J Appl Sport Psychol. (2010) 22(4):486–99. 10.1080/10413200.2010.507497

[25] Zhou Y Wang K Chen S Zhang J Zhou M. The actor, partner, similarity effects of personality, and interactions with gender and relationship duration among Chinese emerging adults. Front Psychol. (2017) 8:1698. 10.3389/fpsyg.2017.01698 29033878 PMC5626872

[26] Marlow SL Lacerenza CN Paoletti J Burke CS Salas E. Does team communication represent a one-size-fits-all approach? A meta-analysis of team communication and performance. Organ Behav Hum Decis Process. (2018) 144:145–70. 10.1016/j.obhdp.2017.08.001

[27] Held M Rieger JW Borst JP. Multitasking while driving: central bottleneck or problem state interference? Hum Factors. (2024) 66(5):1564–82. 10.1177/00187208221143857 36472950 PMC10943624

[28] Li W Li R Xie X Chang Y. Evaluating mental workload during multitasking in simulated flight. Brain Behav. (2022) 12(4):e32489. 10.1002/brb3.2489 PMC9014989 35290712

[29] Monsell S. Task switching. Trends Cogn Sci (Regul Ed). (2003) 7(3):134–40. 10.1016/S1364-6613(03)00028-7 12639695

[30] Kiesel A Steinhauser M Wendt M Falkenstein M Jost K Philipp AM. Control and interference in task switching—a review. Psychol Bull. (2010) 136(5):849–74. 10.1037/a0019842 20804238

[31] Visser L Pat-El R Lataster J Van Lankveld J Jacobs N. Beyond difference scores: unlocking insights with polynomial regression in studies on the effects of implicit-explicit congruency. Psychol Belg. (2024) 64(1):5–23. 10.5334/pb.1246 38618168 PMC11012099

[32] Schönbrodt FD Humberg S Nestler S. Testing similarity effects with dyadic response surface analysis. Eur J Pers. (2018) 32(6):627–41. 10.1002/per.2169

